# Bioreduction of precious and heavy metals by *Candida* species under oxidative stress conditions

**DOI:** 10.1111/1751-7915.13364

**Published:** 2019-01-07

**Authors:** Abel Moreno, Nicola Demitri, Estela Ruiz‐Baca, Arturo Vega‐González, Maurizio Polentarutti, Mayra Cuéllar‐Cruz

**Affiliations:** ^1^ Instituto de Química Universidad Nacional Autónoma de México Ciudad Universitaria Av. Universidad 3000 Ciudad de México 04510 México; ^2^ Elettra – Sincrotrone Trieste S.S. 14 km 163.5 in Area Science Park 34149 Basovizza – Trieste Italy; ^3^ Facultad de Ciencias Químicas Universidad Juárez del Estado de Durango Av. Veterinaria S/N 34120 Durango México; ^4^ Departamento de Ingenierías Química Electrónica y Biomédica División de Ciencias e Ingenierías Universidad de Guanajuato Campus León Guanajuato México; ^5^ Departamento de Biología División de Ciencias Naturales y Exactas Universidad de Guanajuato Campus Guanajuato, Noria Alta S/N, Col. Noria Alta C.P. 36050 Guanajuato México

## Abstract

The aim of the present work was to evaluate whether *Candida* species can reduce both precious and toxic pure metals from the respective molecular ions. From these results, the nanoparticles formed were studied using scanning electron microscopy with energy‐dispersive spectroscopy, Raman spectroscopy, X‐ray fluorescence spectroscopy and synchrotron radiation. Our results showed that the metal ions were reduced to their corresponding metallic nanoconglomerate or nanoparticles by *Candida* species. This is the first report on how yeasts of this genus are capable of achieving homeostasis (resilience) in the presence of metal ions of both precious and toxic metals by reducing them to a metallic state.

## Introduction

For thousands of years, metallic minerals have been of great value to mankind, since they are widely used from tools making to the construction of buildings and houses, and even to generate energy and to manufacture jewellery (Cuéllar‐Cruz *et al*., 2017). Among these minerals we find precious metals such as gold and silver, as well as other widely used metals, like mercury, lead, copper, nickel, iron, aluminium. Nowadays, the obtainment of pure metals by high‐cost mineral treatments such as pyrometallurgical and hydrometallurgical processes (Marchant, 1985) has been substituted by less expensive biological procedures known as bioleaching (Vera *et al*., 2013). These procedures make use of a large diversity of microorganisms such as bacteria, archae and yeasts (Kelly *et al*., 1979; Hutchins *et al*., 1986; Norris and Parrott, 1986; Wiegel and Ljungdahl, 1986; Biryuzova *et al*., 1987; Kelly and Harrison, 1989; Rawlings and Kusano, 1994; Clark and Norris, 1996; Karamushka and Gadd, 1999; Norris *et al*., 2000; Brandl *et al*., 2001; Vera *et al*., 2013; Madrigal‐Arias *et al*., 2015) resulting in the isolation of macroscopic forms of minerals. Contrary to macroscopic metals, nanoparticles (NPs) exhibit physical and chemical characteristics such as optical, electrical, magnetic, colligative and catalytic properties, that depend from the form, size and method of isolation (Lu *et al*., 2013). Gold and silver NPs (AuNPs, AgNPs) are particularly important as some of their properties have allowed their use as therapeutic alternatives (Mandal *et al*., 2006; Asharani *et al*., 2010, 2011). AuNPs, for instance, are potentially useful as carriers of therapeutic agents and in gene therapy and also as a phototherapeutic aid in the early detection, diagnoses and treatment of cancer (Paciotti *et al*., 2004, 2006; Chen *et al*., 2008; Jain *et al*., 2008). In the same line, AgNPs have been used as antimicrobial compounds, topic creams and as anticancer products (Firdhouse and Lalitha, 2015). Several laboratories have recently focused their interest on the obtainment of NPs of toxic metals such as lead and mercury by biological synthesis from their molecular ions present in waste waters from the mining industry to prevent further contamination of water bodies and arable lands. Microorganisms such as bacteria, yeasts and filamentous fungi are able to synthesize these nanostructures (Mandal *et al*., 2006; : Kharissova *et al*., 2013), which tend to oxidize by giving up their electrons to reduce the metal to zero valence.

We have recently used species the genus *Candida* that are able to form nanocrystals of lead sulphide, mercury and cadmium (Cuéllar‐Cruz *et al*., 2017). However, it has not been evaluated whether these yeasts are capable of synthesizing micro‐ or nanoparticles (MPs, NPs) of precious and heavy metals. The aim of the present work was to evaluate the capacity of *C. albicans, C. dubliniensis* and *C. glabrata* to synthesize NPs of precious metals (gold and silver) or heavy metals (mercury and lead), by reducing their corresponding molecular ions. The formation of the metallic NPs was carried out with the three *Candida* species, for which the yeasts were grown in YPD (yeast extract, 1%; peptone and glucose, 2%) in the presence of 1.0 mM of the metal ions and 100 mM of hydrogen peroxide (H_2_O_2_) for 48 h at 28°C. In this case, the addition of an oxidizing agent was necessary, because *Candida* species are not capable of reducing the cations of precious or heavy metals in non‐oxidizing conditions (Cuéllar‐Cruz *et al*., 2017). The NPs formed were evaluated using scanning electron microscopy with energy‐dispersive spectroscopy (SEM‐EDS), Raman spectroscopy, X‐ray fluorescence spectroscopy and synchrotron radiation. Our results showed that the metal ions of Au^3+^, Ag^+^, Pb^2+^ or Hg^2+^ are reduced to their corresponding NPs by *Candida* species. This is the first report that shows that yeasts of this genus are able to achieve a homeostasis (resilience) in the presence of metal ions of both precious and toxic metals, by reducing them to a metallic state. These data indicate that *Candida* species have developed mechanisms that enable them to adapt to different habitats.

## Results

### *Candida* species tolerate precious and heavy metals

**Figure 1:**
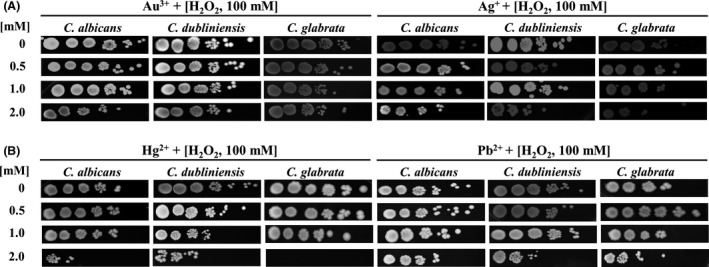
Susceptibility assays in *C. albicans*,* C. dubliniensis* and *C. glabrata* in the presence of (A) precious metals Au^3+^ or Ag^+^, or (B) heavy metals Hg^2+^, Pb^2+^. Cultures of treated cells with any of the precious or heavy metals were under oxidizing conditions. Control cells were not treated with any cation.

*Candida* cells were exposed to different concentrations of each of the metals in the presence of 100 mM of the oxidizing agent, hydrogen peroxide (H_2_O_2_). The chosen concentration of the oxidizing agent does not alter the cellular viability of the *Candida* species used (Cuéllar‐Cruz *et al*., 2008; Ramírez‐Quijas *et al*., 2015). As observed in Fig. 1A, in the presence of Au^3+^ or Ag^+^ the cells exposed to oxidizing conditions are able to tolerate up to 2.0 mM of these elements. *C. albicans* and *C. dubliniensis,* in the presence of Hg^2+^, at a concentration of 2.0 mM are susceptible. Nonetheless, *C. glabrata* at this concentration is unable to survive (Fig. 1B). Regarding Pb^2+^, the three *Candida* species are able to tolerate up to a concentration of 2.0 mM (Fig. 1B). Based on these results, we decided to assess whether *C. albicans*,* C. dubliniensis* and *C. glabrata* could reduce the cations of both the precious and the heavy metals up to 1.0 mM concentration (Fig. 1). At this concentration, *Candida* cells are viable. These results agree with those published results from different groups that have shown that yeasts possess mechanisms that have allowed them to survive in different habitats, from the human body to soil and water contaminated with precious or heavy metals (Hagler and Mendonca‐Hagler, 1981; Suihko and Hoekstra, 1999; Lopez‐Archilla *et al*., 2004; Harrison *et al*., 2006; Cuéllar‐Cruz *et al*., 2017).

### Biosynthesis of precious metal nanoparticles: gold and silver

**Figure 2:**
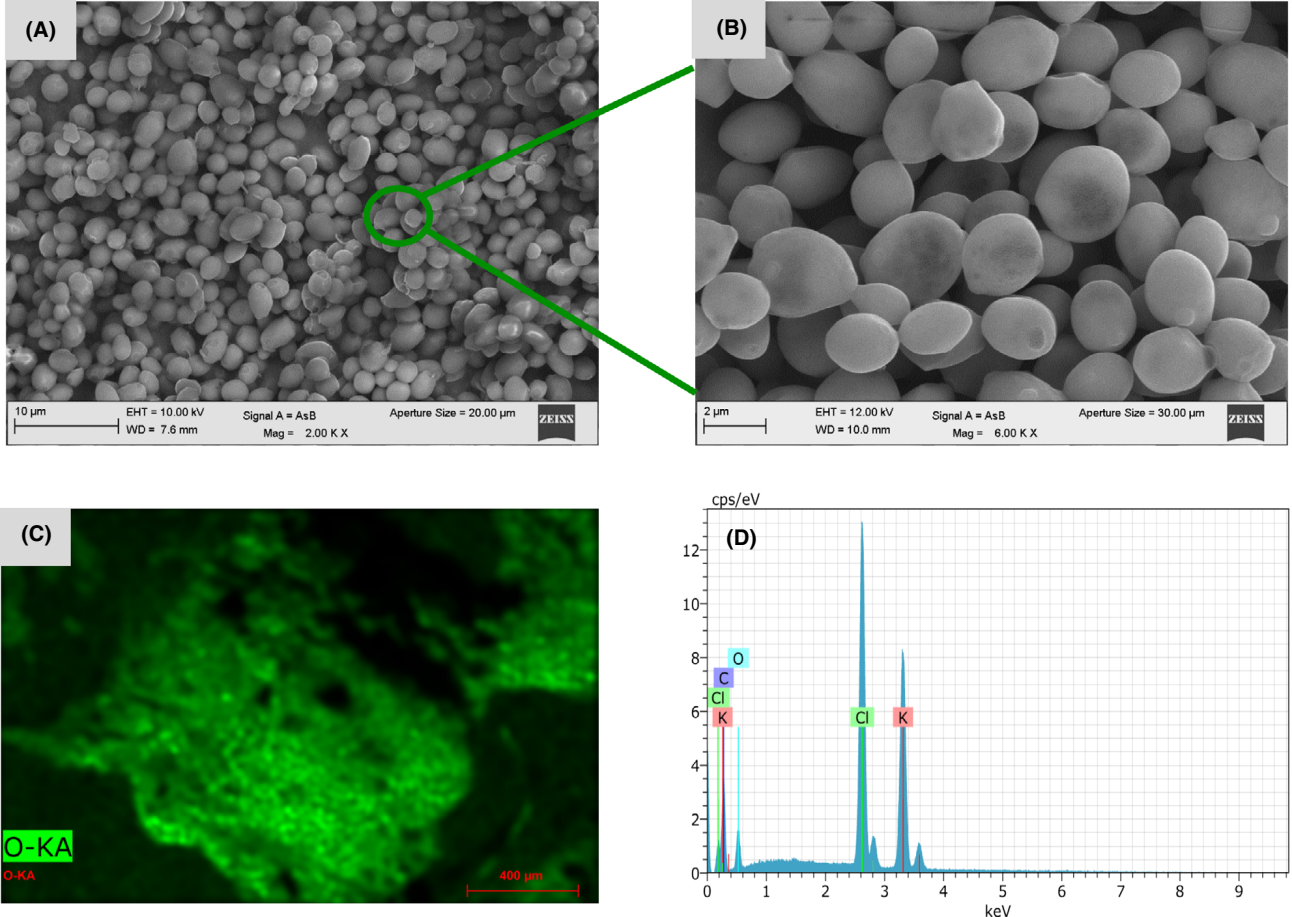
Control cells of *Candida* species in absence of precious or heavy metals. A, B. The control cells were analysed by means of SEM as described in the *methods* section. Scale bar is indicated in each photomicrograph to show the size of the cells. C, D. Qualitative analysis of the elements present in the control cells by means of energy‐dispersive spectroscopy (EDS).

**Figure 3:**
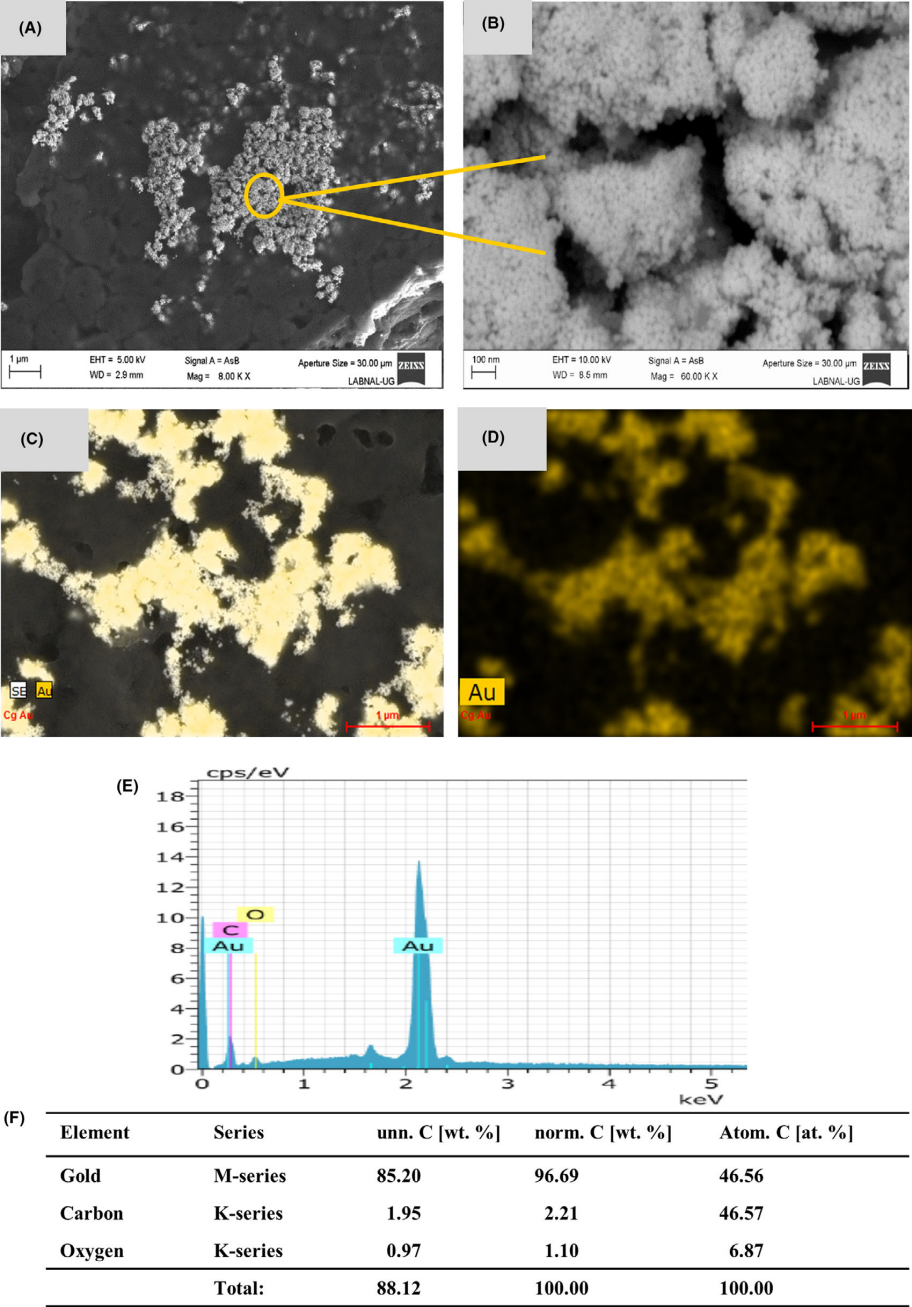
Formation of gold nanoparticles formed by the *Candida* species in the presence of Au^3+^. A, B. The AuNPs were analysed by means of SEM as described in the *methods* section. Scale bar is indicated in each photomicrograph. Yellow arrows and circle indicate the NPs formed. C–E. Energy‐dispersive spectroscopy (EDS) qualitative analysis of the elements present in the AuNPs. F. Percentage of the element present in the sample. As shown in the EDS plot, the NPs are formed from the reduced metal.

**Figure 4:**
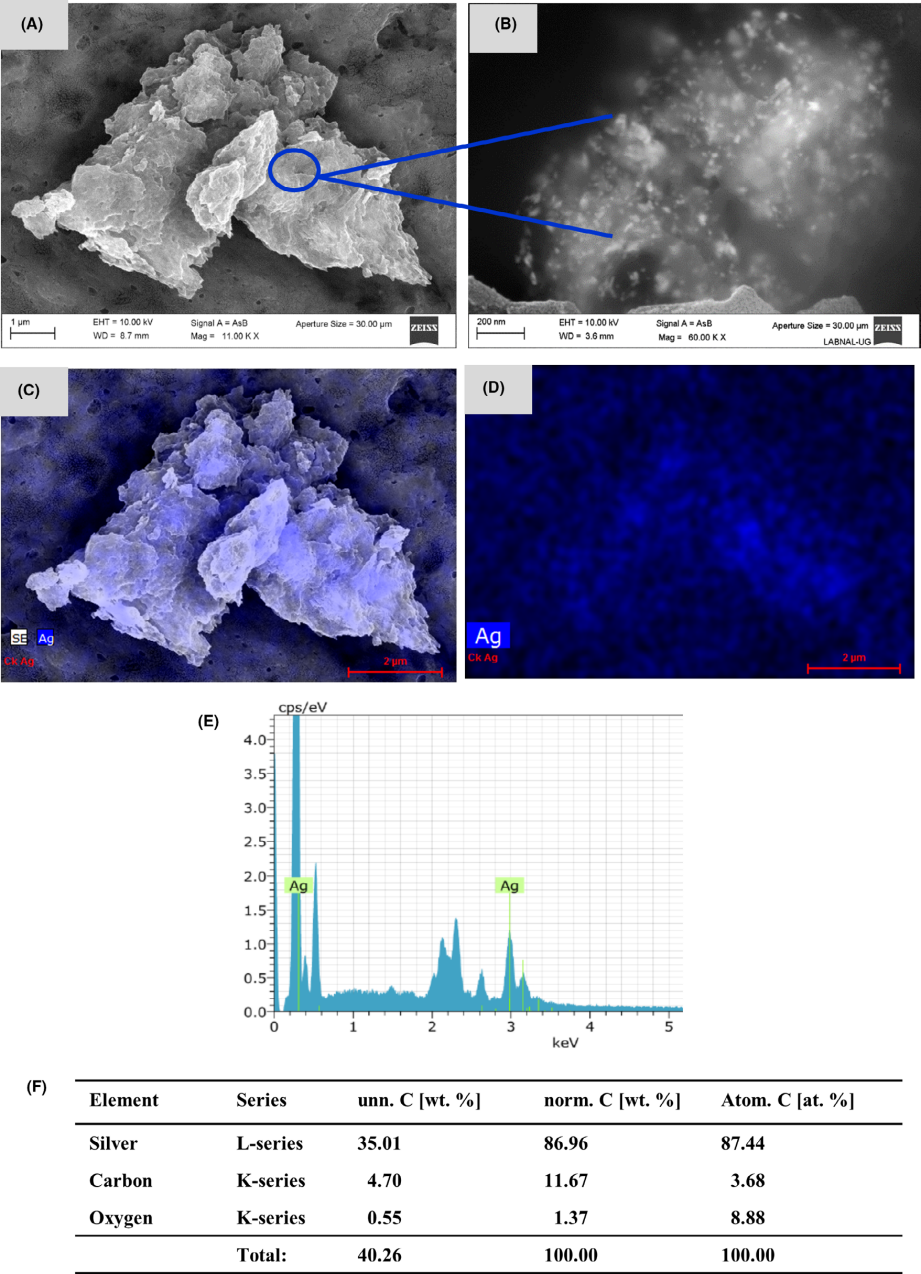
Formation of silver nanoparticles formed by the *Candida* species in the presence of Ag^+^. A, B. The AgNPs were analysed by means of SEM as described in the *methods* section. Scale bar is indicated in each photomicrograph. Blue arrows and circle indicate the silver nanoconglomerates. C–E. Qualitative analysis of the elements present in the Ag nanoconglomerates through energy‐dispersive spectroscopy (EDS). F. Percentage of the element present in the sample. As shown in the EDS plot, the nanoconglomerates are formed from the reduced metal.

In order to evaluate whether the *Candida* species were able to synthesize NPs from the gold, or silver ions in solution, 100 mM of H_2_O_2_ were added to favour oxidizing conditions. In this way, the ions in solution were reduced by each of the *Candida* species. In addition to the oxidizing conditions, it has been reported that those microorganisms that perform the synthesis process are characterized by growing in acidic environments (Vanderrest *et al*., 1995; Muhlschlegel and Fonzi, 1997; Gupta *et al*., 2000). In the case of the *Candida* species, this is shown below as they reach an acidic pH (Cuéllar‐Cruz *et al*., 2017). As illustrated in Fig. 2, control cells in the presence of only the oxidant agent do not form NPs of any type. Notwithstanding, cells treated with Au^3^ or Ag^+^ showed scarce or a lack of extracellular particles (Fig. S1). Therefore, in order to perform an adequate analysis of the metallic particles of these elements it was necessary to subject the cells to lysis. Representative microphotographs, taken through SEM, of the obtained results in the three *Candida* species in the presence of Au^3+^ are shown in Fig. 3 and S2. *Candida* cells, in the presence of Au^3+^, can reduce this cation to gold NPs (AuNPs, see Fig. 3 and S2). As seen in Fig. 3A, B and S2 the Au^0^ nanoparticles (AuNPs) are grouped in clusters. Under higher magnification, we can see the AuNPs, which have a completely spherical shape (Fig. 3B). Analysing the samples under SEM, AuNPs clusters were found in all the analysed fields, which shows that the *Candida* species have the ability to efficiently reduce Au^3+^ to Au^0^. Additionally, to corroborate that the AuNPs observed through SEM corresponded to Au^0^, the analysis of the elements present in the sample was carried out by means of EDS. Additionally, the percentage of these elements present in the sample was determined. As shown in the representative figure, in the analysed AuNPs, only Au^0^ was found (Fig. 3C–F). However, in order to confirm this result, the AuNPs were afterwards analysed through X‐ray fluorescence and synchrotron radiation. The very small percentage of carbon and oxygen shown by the EDS analysis is due to the sample processing to be analysed by SEM‐EDS and/or due to the cell residues present in the samples (Fig. 3E, F). After finding that *Candida* species were able to reduce molecular ions of gold, we wondered if these microorganisms could also reduce silver. The three species of *Candida* were exposed to the Ag^+^ ion under the same conditions as we did for gold. Interestingly enough, when observing the samples through SEM, we found that, unlike gold (which formed clusters), silver formed ‘nanorocks’ or ‘nanoconglomerates’ (Fig. 4A and S3). In a microphotograph at a higher magnification, it is remarkable to see how these nanorocks or nanoconglomerates of silver nanoparticles (AgNPs) were formed (Fig. 4B). These AgNPs appear in the form of elongated bars (Fig. 4B), but are smaller than the AuNPs (Fig. 3A, B). To corroborate that the nanorocks or nanodeposits effectively corresponded to elemental silver, the analysis was carried out by EDS. The analysis revealed that they actually corresponded to Ag^0^ (Fig. 4C–F). These data were then confirmed with X‐ray fluorescence and synchrotron radiation. Through EDS applied on these samples, we also found a very small percentage of carbon and oxygen due, as mentioned before, to the processing and/or cell residues present in the samples (Fig. 4E, F).

### Obtaining heavy metal particles: mercury and lead

**Figure 5:**
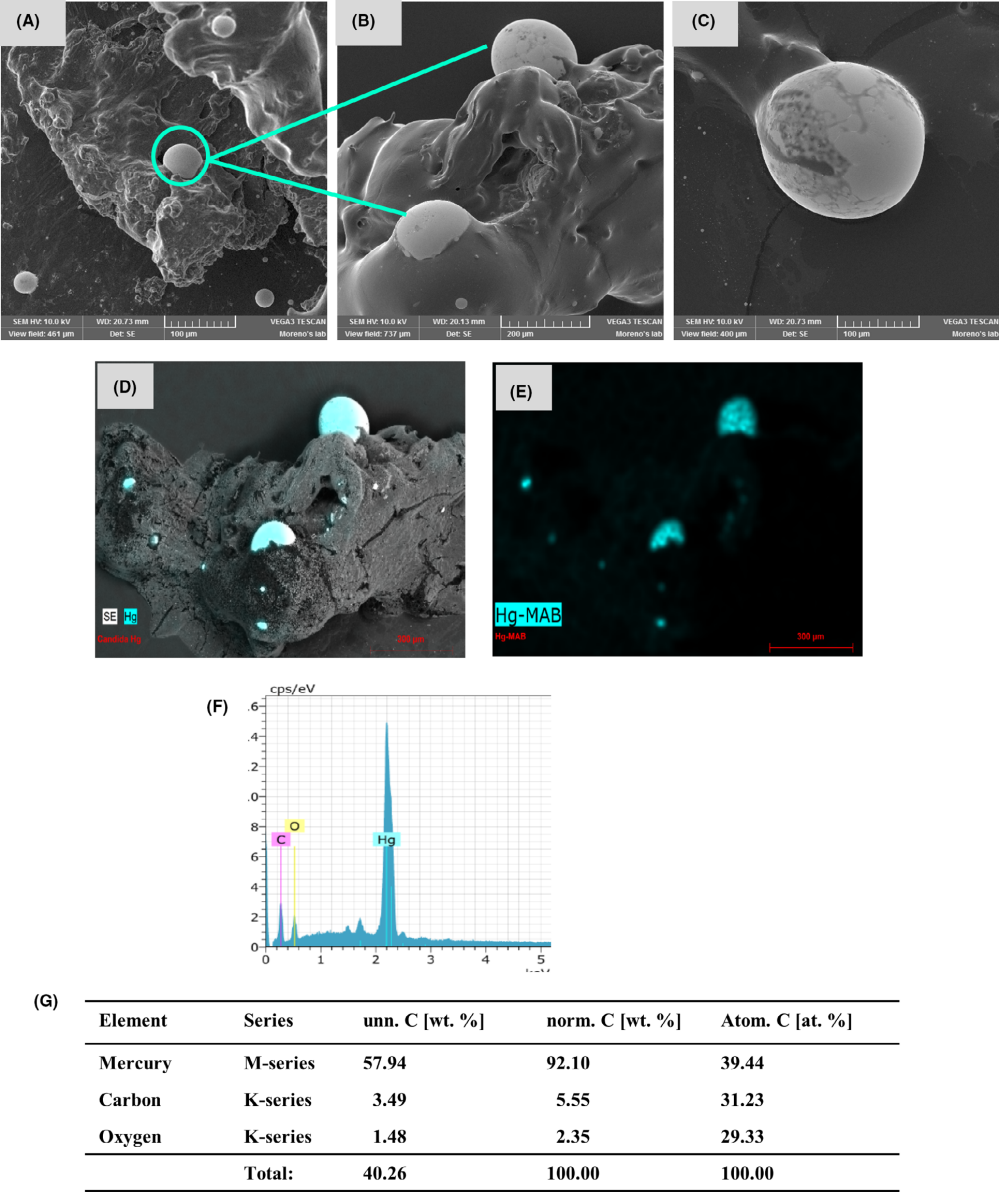
Formation of mercury drops by the *Candida* species in the presence of Hg^2+^. A–C. The Hg drops were analysed by means of SEM as described in the *methods* section. Scale bar is indicated in each photomicrograph. Green arrows and circle indicate the drops formed. D–F. Qualitative analysis of the elements present in the HgNPs through energy‐dispersive spectroscopy (EDS). G. Percentage of the element present in the sample.

In order to evaluate whether the *Candida* species, apart from reducing precious metals, could also reduce toxic metals such as lead and mercury, the cells of these yeasts were exposed to Pb^2+^ or Hg^2+^. The treated cells were observed through SEM. *Candida* cells exposed to Hg^2+^, a minimal presence of extracellular microdrops was found (Fig. S1). The largest concentration of these microdrops was found intracellularly; thus, the cells were lysed. Observing the microphotographs of the lysed samples exposed to Hg^2+^, Hg^0^, we found micrometre diameter droplets in practically the entire sample (Fig. 5A–C and S4). The mercury microdrops displayed the cohesion property characteristic of Hg in liquid form, when we placed close to one another these drops, both drops of liquid mercury bound to each other forming a larger drop (Fig. 5C). The drops formed were analysed through EDS, which corroborated that they were formed of Hg^0^ (Fig. 5D, E, F, G and S4).

**Figure 6:**
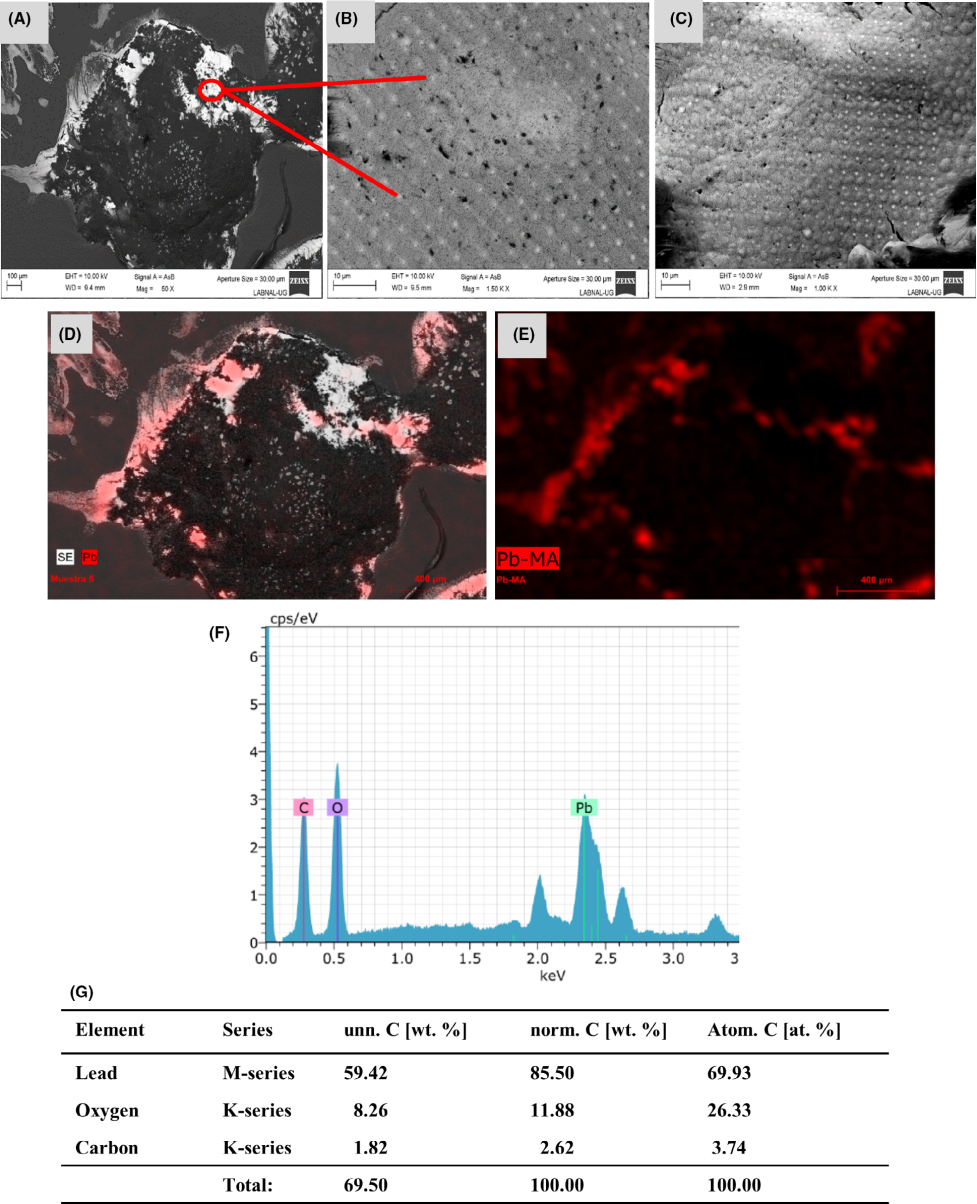
Formation of lead nanoparticles formed by the *Candida* species in the presence of Pb^2+^. A–C. The PbNPs were analysed by means of SEM as described in the *methods* section. Scale bar is indicated in each photomicrograph. Red arrows and circle indicate the NPs formed. D–F. Qualitative analysis of the elements present in the PbNPs through energy‐dispersive spectroscopy (EDS). G. Percentage of the element present in the sample.

When observing cells exposed to Pb^2+^, we found scarce or a lack of extracellular metallic particles (Fig. S1); thus, we decided to disrupt the cells. In the case of the lysed samples from the cells exposed to Pb^2+^, ‘nanodeposits’ were found (Fig. 6A and S5). Looking closely at these nanodeposits and taking microphotographs it was observed that the particles of Pb^0^ were perfectly ordered, forming perfect squares with a particle of Pb^0^ (Fig. 6B, C) in each vertex. These lead‐treated samples were analysed through EDS, which revealed that the nanodeposits were formed by Pb^0^ (Fig. 6D–G). The low percentage of oxygen and carbon present in both mercury and lead samples (Figs 5F, G and 6F, G) is due to the same causes mentioned for gold and silver.

### Characterization of Au, Ag, Pb and Hg by Raman spectroscopy and X‐ray powder diffraction (XRPD)

Gold, silver and lead NPs and mercury drops synthesized by *C. albicans*,* C. dubliniensis* and *C. glabrata* were characterized by Raman spectroscopy and XRPD as described in the *materials and methods* section, based on the fact that NPs exhibit unique physicochemical properties that depend on their shape and size (Poulose *et al*., 2014; Firdhouse and Lalitha, 2015). These characteristics of the nanometric dimension are due to the surface/volume ratio and quantum confinement of NPs, which are displayed as a consequence of an increase in the space between the levels of electronic energy due to a decrease in particle size (Daniel and Astruc, 2004). Raman spectroscopy helps to identify molecules through spectral information, which is considered a molecular fingerprint. This technique has been widely used for the identification of NPs of different chemical composition (Lu *et al*., 2013; Chen *et al*., 2017). Raman spectroscopy, as observed in Table 1, when used for the characterization of AuNPs, revealed two peaks at 589 and 1102 nm wavelengths, which are close to those previously reported for gold nanospheres (Kalmodia *et al*., 2013). For AgNPs, three peaks at 437, 1594 and 1646 nm were identified, corresponding to values essentially similar to those found for silver nanoparticles (Lu *et al*., 2013; Tu and Chung, 2017). These results strongly indicate that *Candida* species are able to synthesize AgNPs. We detected three peaks for NPs of lead at 445, 1096 and 4396 nm. For mercury drops, three peaks at 254, 3843 and 4168 nm were also detected. However, due to the size of AuNPs and AgNPs, the intensity of the peaks was not highly enough to be characterized by Raman spectroscopy. This led us to corroborate these results by synchrotron radiation setting up a powder diffraction in capillary tubes to collect data from each *Candida* species.

**Table 1:** Identification of the chemical composition of the nanocrystals formed by Raman

Metal	λ cm^−1^	*Candida* specie
*Candida* without metal	ND	*C. albicans*,* C. dubliniensis*,* C. glabrata*
Au^0^	589, 1102	*C. albicans*,* C. dubliniensis*,* C. glabrata*
Ag^0^	437, 1594, 1646	*C. albicans*,* C. dubliniensis*,* C. glabrata*
Pb^0^	445, 1096, 4396	*C. albicans*,* C. dubliniensis*,* C. glabrata*
Hg^0^	254, 3843, 4168	*C. albicans*,* C. dubliniensis*,* C. glabrata*

NA, Not applicable; ND, no signal detected.

The samples and data obtained were analysed as described in the *methods* section.

**Figure 7:**
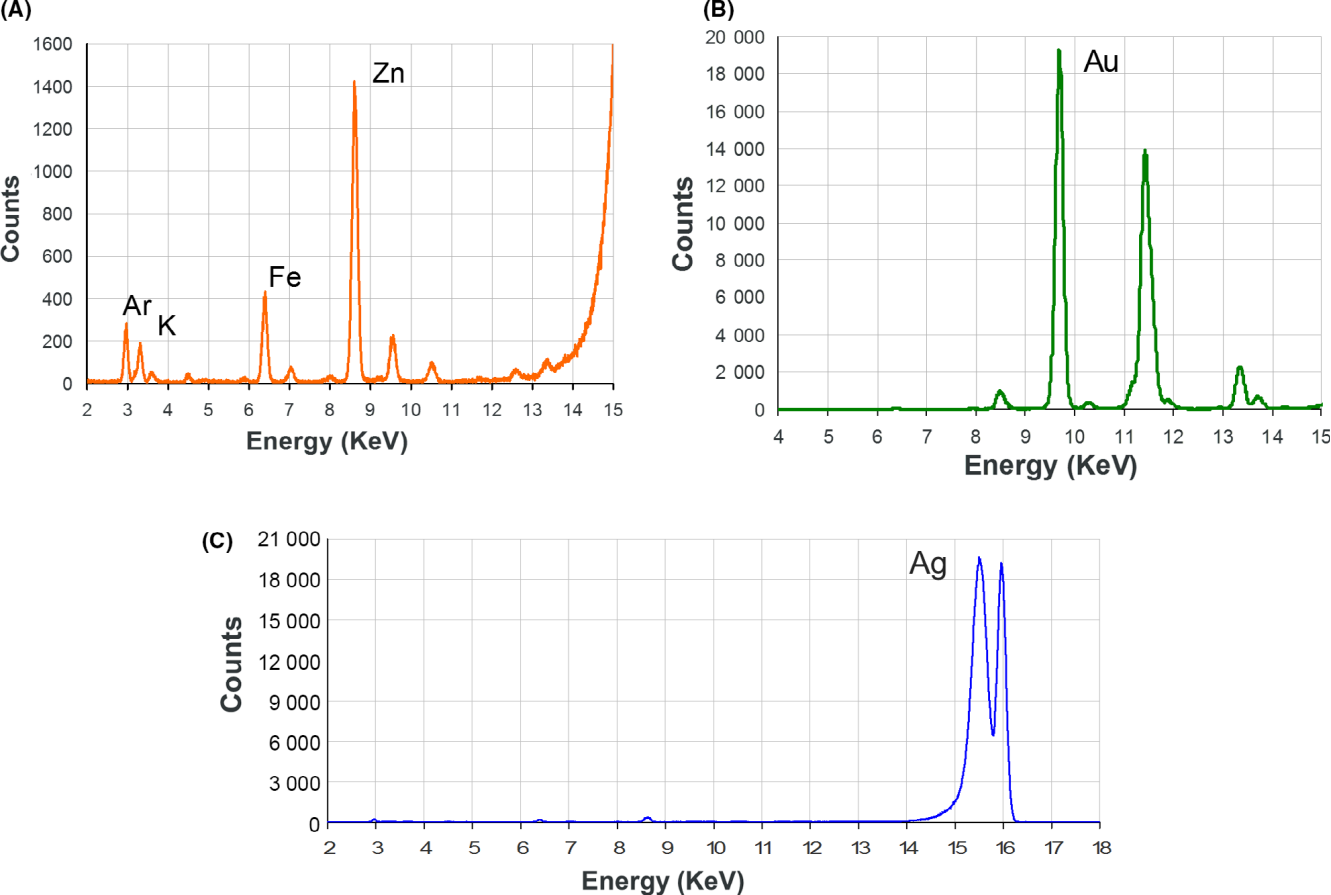
Fluorescence peaks interpretation for: (A) *Candida* blank sample packed in capillary (16 keV excitation energy) – elements labelled on corresponding K_α_ lines. (B) Peaks for a *Candida* gold loaded sample, belong to Au^0^; element is labelled on its L_α_ lines. (C) Peaks for a *Candida* silver loaded sample, belong to Ag^0^; element is labelled on its L_α_ lines.

X‐ray fluorescence spectroscopy confirmed the presence of gold in *C. albicans*,* C. dubliniensis* and *C. glabrata*, with superimposable patterns among different cell lines (Fig. 7B and Table 2). Nonetheless, endogenous zinc and other common metals (K, Fe) were found, even in blank samples (Fig. 7A). Spectra obtained from silver loaded samples (Fig. 7C) are not informative due to heavy air absorption of emission peaks at photon energies below 3.5 keV and peak overlaps with widespread bioelements like potassium, calcium, chlorine and atmospheric argon.

**Table 2:** Elements detected in sample fluorescence spectra using 16 keV excitation energy

	*C. albicans*	*C. glabrata*	*C. dubliniensis*
Capillary blank	[Ar], K, Fe, [Cu], Zn, [Pb], [Br]		
Au loaded	[Ar], [K], [Fe], Zn, **Au**		
Ag loaded^a^	[Ag‐Ar], [K], [Fe], Zn, **Ag**		

Elements reported in square parenthesis seem present as traces and could be due to environmental contamination – bolded symbols refer species estimated as more abundant from elemental analysis and, qualitatively from fluorescence intensities.

^a^Ag peaks cannot be clearly assigned, due to heavy air absorption of photons below 3.5 keV and peak overlaps with widespread bioelements like K, Ca, Cl and Ar.

**Figure 8:**
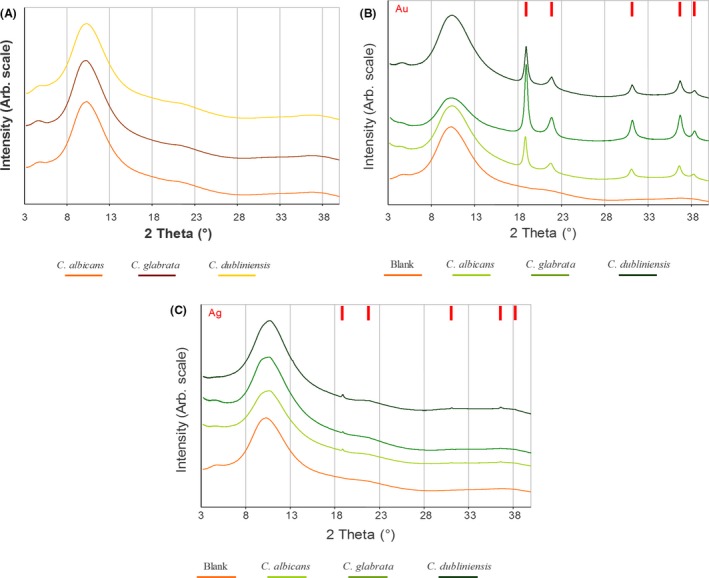
X‐Ray powder patterns of Au^0^ or Ag^0^. A. *Candida* cells not exposed to heavy metals (blanks). B. *Candida* cells exposed to gold. Red bars represent expected positions of ccp *F m3m* Au (Wyckoff, 1963). C. *Candida* cells exposed to silver. Red bars represent expected positions of ccp *F m3m* Ag (Wyckoff, 1963). All samples had diffraction peaks at 16 keV, calculated using CCDC Mercury (Macrae *et al*., 2008). Patterns are vertically shifted for clarity.

X‐ray powder patterns collected from *Candida* cells not exposed to heavy metals (Fig. 8A) show broad peaks that agree with previous data published for glucans extracted from cell walls (Lowman *et al*., 2014) and ‘poorly ordered’ lipidic phases (giving a broad peak at ~4.3 Å; Tyler *et al*., 2014). Blank background patterns of the three *Candida* species analysed are superimposable.

The presence of heavy atoms in the cells introduces sharper signals that belong to crystalline metallic nanoparticles. For AuNPs (Fig. 8B), sharp signals match nicely the peak positions expected for crystalline cubic closest packed (ccp) metallic gold *F m3m* phase (Wyckoff, 1963) samples from different cells lines are equivalent. The same result is found in cell lines loaded with silver, where the crystalline silver *F m3m* phase is found (Wyckoff, 1963) in all the cell lines considered (Fig. 8C). Taken together, these results indicate that in the presence of an oxidant, such as H_2_O_2_, *Candida* species are able to reduce ions of both precious and heavy metals (Figs 3, 4, 5, 6).

## Discussion

Nanoparticles can be synthesized by chemical and biological methods, the latter using mostly bacteria, fungi and plants. In both methods, conditions are optimized to carefully control the size and form to obtain monodisperse nanoparticles of identical crystalline structure and chemical composition. Numerous studies have found that many microorganisms can synthesize NPs (Li *et al*., 2011; Zhang *et al*., 2011; Moghaddam *et al*., 2015). These works show that each microorganism is able to synthesize either a corresponding metal sulphide nanocrystal or metallic NPs but not both (Zhang *et al*., 2011). The *Candida* species can synthesize nanocrystals of lead sulphide, mercury or cadmium (Cuéllar‐Cruz *et al*., 2017) and reduce cations of precious or heavy metals to the corresponding NPs (Figs 3, 4, 5, 6, 7, 8, Tables 1 and 2). The latter indicates that these microorganisms have developed specific mechanisms under specific conditions, which allow them to achieve homeostasis with the metals to which they are exposed, and thus adapt to the different habitats that they encounter. *Candida* species have been identified in different habitats from soils and water contaminated with heavy metals to organs or bloodstream of humans (Hagler and Mendonca‐Hagler, 1981; Suihko and Hoekstra, 1999; Lopez‐Archilla *et al*., 2004; Cuéllar‐Cruz *et al*., 2017). A condition required in the formation of metallic NPs, in addition to an oxidizing condition, is an acidic medium (Agnihotri *et al*., 2009). We added H_2_0_2_ to the culture medium to achieve an oxidizing condition. It is important to remark that without adding the oxidizing agent, the *Candida* species are not able to reduce the ions of precious or toxic metals. Interestingly, in the absence of H_2_0_2_, *Candida* synthesizes sulphur nanocrystals of the corresponding metal (Cuéllar‐Cruz *et al*., 2017; Moreno *et al*., 2019). This shows that *Candida*, unlike other fungi, has specific mechanisms to synthesize nanocrystals or NPs, a characteristic of *Candida* species that make them an excellent model to use as producers of nanocrystals of lead sulphide, mercury or cadmium (Cuéllar‐Cruz *et al*., 2017) or of NPs of gold, silver or lead, as well as mercury drops (Figs 3, 4, 5, 6, 7, 8).

Regarding AuNPs, the microorganisms widely used to synthesize these NPs are bacteria, where *Bacillus subtilis 168* has been reported to be able to reduce Au^3+^ to AuNPs with a size ranging between 5 and 25 nm (Southam and Beveridge, 1996). *Halomonas salina* is another bacterium that synthesizes AuNPs, only that the morphology of these NPs varies according to whether it is found in acid or basic medium. In fungi, *Verticillum* sp. are reported to be able to synthesize AuNPs, with the disadvantage that the NPs must be extracted from the interior of the fungal biomass (Zhang *et al*., 2011). Extremophilic yeast has been reported to synthesize AuNPs that show irregular shapes (Mourato *et al*., 2011). In the case of *Candida* species, they have advantages over the bacteria and fungi described above, since *Candida* synthesizes fully spherical, uniform, and stable AuNPs (Fig. 3 and S2).

AgNPs are of special interest due to different uses in medicine and as a microbicide. In the synthesis of AgNPs, it has been described that most of the microorganisms are not capable of producing them due to their toxicity. However, bacteria that are resistant to silver have been identified (Silver, 2003). The bacterium *Pseudomonas stutzeri* AG259 produces AgNPs with a size ranging between 35 and 46 nm. Another bacterium that synthesizes AgNPs is *Idiomarina* sp., which produces NPs with an average size of 26 nm (Slawson *et al*., 1994). In yeasts, *Pichia capsulta* is reported to be able to synthesize AgNPs in an extracellular manner (Srivastava and Kowshik, 2015). Another fungus that has been reported to be able to synthesize AgNPs is the filamentous fungus *Verticillium* sp. This fungus is able to synthesize spherical intracellular AgNPs with an average size of 25 nm, but the disadvantage is that they must be recovered from the fungal mass. Halophilic fungi such as *Thraustochytrium* sp. and *Aspergillus niger* have also been reported to synthesize AgNPS (Mandal *et al*., 2006).

Another element of interest in this study was Hg^2+^, which was evaluated to determine whether it could be reduced by *Candida* species. The fact that mercury droplets are formed by *Candida* species makes it a rather interesting finding as mercury is toxic to mammals and microorganisms (Clarkson, 1997; Diamond and Zalups, 1998; Westwater *et al*., 2002). However, *Candida* cells were not only able to survive in the presence of Hg^2+^ ions, but they were also able to reach homeostasis with these toxic ions by reducing them and forming the Hg^0^ drops (Fig. 5 and S4). It has been reported in *Saccharomyces cerevisiae* that the toxicity of mercury is due to the binding of mercury to thiol‐containing compounds, such as glutathione, resulting in oxidative stress (Kungolos *et al*., 1999; Miura *et al*., 1999). Probably, the fact that mercury generates oxidative stress, coupled to the stress that H_2_O_2_ had already generated in the sample, enables the *Candida* species to reduce the Hg^2+^ ions to form the Hg^0^ drops (Fig. 5). It has been recently reported that *Candida* species can form mercury nanocrystals (Cuéllar‐Cruz *et al*., 2017), but it has not been shown whether these yeasts are capable of producing HgNPs or not.

Finally, we also decided to evaluate Pb^2+^. In the case of the PbNPs synthesized by *Candida* (Fig. 6 and S5) this is the first report, to our knowledge, where the reduction of Pb^2+^ to Pb^0^ has been reported in a microorganism. In other studies, the formation of lead sulphide nanocrystals has been reported in both bacteria and yeasts (Ingale and Chaudhari, 2013; Cuéllar‐Cruz *et al*., 2017) but not the synthesis of PbNPs.

**Figure 9:**
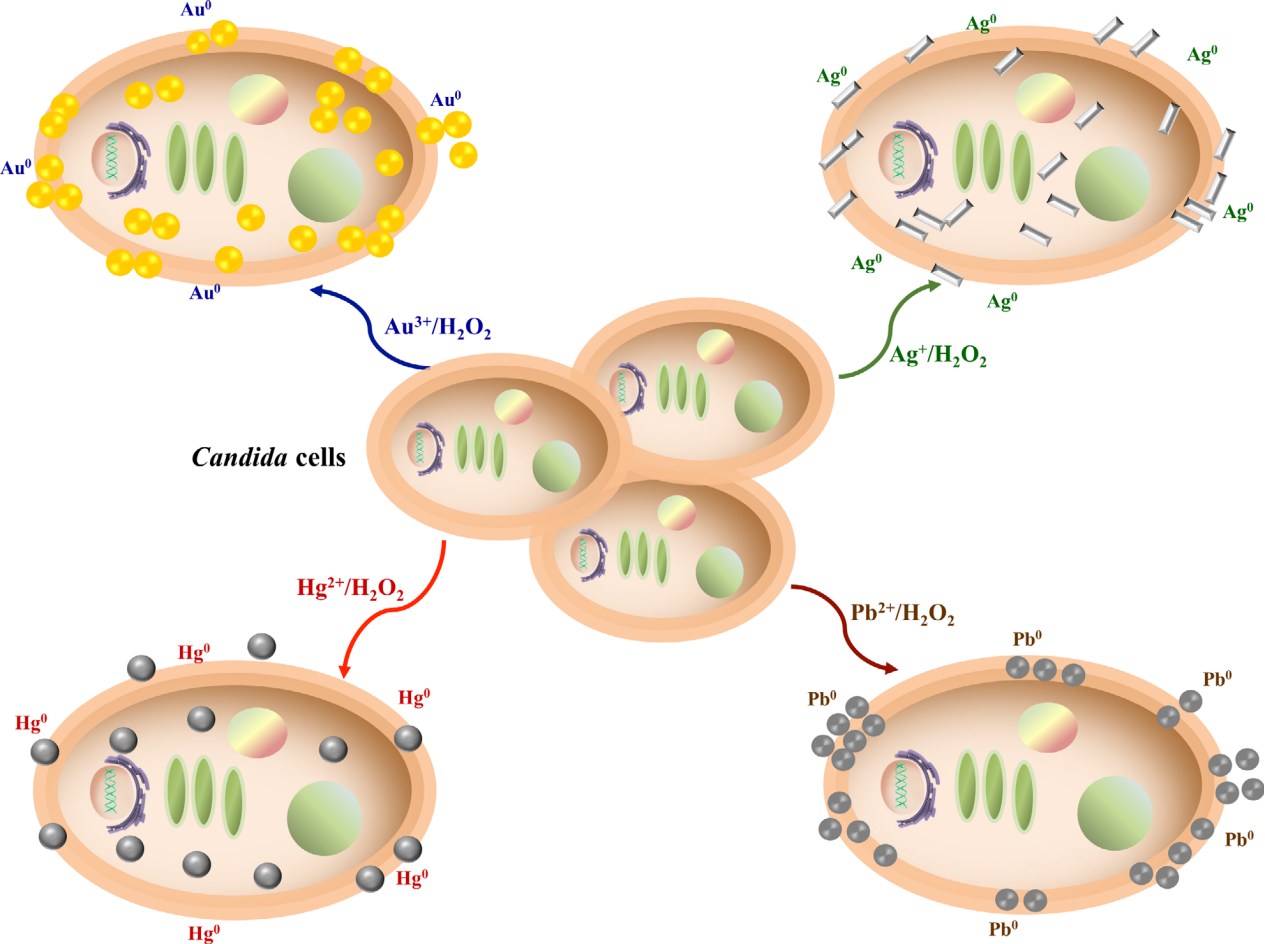
Proposed mechanism through which *Candida* species synthesize NPs of gold, silver and lead, as well as mercury drops.

Although the mechanism of biological synthesis of NPs has not been fully elucidated, fungi in general have several characteristics that are advantageous for the synthesis of metal NPs. It has been shown that fungi are able to synthesize NPs by two routes, intracellular and extracellular, through reduction by enzymes (Moghaddam *et al*., 2015; Cuéllar‐Cruz *et al*., 2017). In the case of *Candida* species, the mechanisms by which they can reduce the cations of precious or heavy metals are probably similar to those reported for other yeasts (Gericke and Pinches, 2006; Agnihotri *et al*., 2009; Sanghi and Verma, 2009; Mourato *et al*., 2011). It is generally proposed that these mechanisms are dependent on enzymes and that the genes for resistance to metals, proteins, peptides, reducing cofactors and organic molecules have significant roles as reducing agents. In addition, they provide NPs with a natural coating, preventing aggregation, stabilizing them for a long time. One proposed mechanism is that metal cations interact with the negatively charged groups of enzymes or polypeptides of the cell wall (CW; Ulberg *et al*., 2010). Another mechanism reported is that, after the ions are trapped in the CW, they are reduced by the enzymes present there (Sastry *et al*., 2003). A third mechanism that has been proposed is that metal ions can diffuse into the cytoplasm and be reduced by the enzymes present in the cytoplasmic membrane and within the cytoplasm (Gericke and Pinches, 2006; Agnihotri *et al*., 2009; Sanghi and Verma, 2009). Even though it has not been described which of the proposed mechanisms is the one followed by microorganisms, it has been reported that the formation of NPs of the different metals is favoured by an acidic pH. A mechanism by which *Candida* species achieve an extracellular acid pH involving the CW is by forming a bond between the metal ions with some component of the wall (Cuéllar‐Cruz *et al*., 2017). An acidic pH is generated because during the formation of the coordinated covalent bond, it can be accompanied by proton dislocation depending on the degree of protonation of the CW (Gupta *et al*., 2000). Another mechanism reported in the maintenance of acid pH is through the *PHR2* gene, which codes for a CW protein involved in the binding of β‐1,3 and β‐1,6 glucans and is expressed in acidic conditions (Muhlschlegel and Fonzi, 1997). In *C. albicans*, it has also been shown that about 500 genes are regulated in response to changes in pH (Bensen *et al*., 2004). Another way that *Candida* species assure an extracellular acid environment is through the membrane ATPase Pma1, which has been reported in *S. cerevisiae* that has a proton export activity (Vanderrest *et al*., 1995). Possibly, by means of these mechanisms, *Candida* species can reduce the metal ions to NPs or drops (Fig. 9). Although the synthesis mechanism of NPs in *Candida* has not been fully elucidated, our working group is working in this direction.

## Conclusions

To our knowledge, this is the first report showing that C*andida* species are able to reduce ions of gold and silver to metallic forms, as well as ions of heavy metals, such as mercury or lead, to their corresponding NPs. Based on these results, we can infer that *Candida* species have developed specific mechanisms that allow them to achieve homeostasis in the presence of metal ions. Therefore, *Candida* can be used in the near future for the bioleaching of different types of water and soil.

## Experimental procedures

### Strains and culture conditions

The strains of *C. albicans*,* C. dubliniensis* and *C. glabrata* used in this study are clinical isolates from the collection of the Department de Microbiology, ENCB‐IPN, Mexico. Yeast strains were cultured on yeast peptone (yeast extract, 1%; peptone and glucose, 2%) and 2% agar was added to solidify the media (Ausubel *et al*., 2003). Obtainment of the precious or heavy metals was induced by the addition to the cell culture of 1.0 mM of hydrogen tetrachloroaurate trihydrate (HAuCl_4_), silver nitrate, lead nitrate or mercury nitrate (all obtained from Sigma‐Aldrich) and 100 mM of hydrogen peroxide (H_2_O_2_).

### Susceptibility assays of *Candida* strains to Au^3+^, Ag^+^, Pb^2+^, Hg^2+^ in oxidizing conditions

*Candida albicans*,* C. dubliniensis* and *C. glabrata* at OD_600 nm_ 1.0 were cultured in 50 ml of YPD medium with 1.0 mM of hydrogen tetrachloroaurate trihydrate (HAuCl_4_), silver nitrate, lead nitrate or mercury nitrate, and 100 mM of hydrogen peroxide (H_2_O_2_), as oxidizing agent, for 48 h at 28°C under constant agitation. Afterwards, cells in stationary stage were removed from the culture medium containing the metals and the oxidizing agent by centrifugation at 10 000 *g* during 5 min. Then, the cells were resuspended in 1 ml of sterile deionized water, obtaining an OD_600 nm_ of 0.5. According to calculations, serial exponential dilutions were made in 96‐well plates. Additionally, cells were seeded by dripping in plates with YPD medium and incubated at 28°C during 48 h. Plates were photographed with the GeneGenius Bioimaging system (Syngene, Cambridge, UK). Experiments were performed in a triplicate. Control samples were not treated with any metal.

### Obtaining precious or heavy metals from lysis of *Candida*

To isolate the precious or heavy metals, yeast protoplasts were obtained as follows: cells of the three *Candida* species treated with each of the different salts of the metal were pelleted by centrifuging at 3500 *g* for 15 min at 4°C, the pellets were washed four times with sterile deionized water, resuspended in water and counted. Aliquots of the cell suspension were resuspended at a final OD_600 nm_ of 1.0 in 1.0 ml of lysis buffer containing 50 mM Tris‐HCl, pH 7.2, 0.8 M sorbitol, 0.8 M KCl, 10 mM MgSO_4_, 15 mM β‐mercaptoetanol and 0.25 mg ml^−1^ lyticase (all reagents from Sigma‐Aldrich, St Louis, MO, USA) and incubated at 37°C. After 3 h, cells were observed with a Zeiss Axiostar microscope (Carl Zeiss, Germany) to assess protoplast formation. This was about 90%. Protoplasts were collected and gently lysed by resuspending in 500 μl of sterile deionized water and the metallic particles formed *in vivo* were pelleted and separated from cellular debris by centrifugation at 120 *g* for 3 min.

### Scanning electron microscopy (SEM)

After the metallic particles were separated from cellular debris, the particles of gold, silver, lead or mercury were thoroughly washed four times with sterile deionized water. Subsequently, the metal particles were lyophilized in a Tousimis auto Samdri 815 critical point dryer for 4 h. The dried samples were covered with a layer of colloidal gold, except for the samples treated with gold. Subsequently, the samples were observed with the scanning electron microscope, model EVO HD15, high definition ZEISS^®^. Finally, the samples were photographed using the secondary electron detector (SE1) at 15 kV under high vacuum conditions and at a working distance of 4 mm. Treated and without treatment *Candida* cells were visualized under the same conditions as the metallic particles.

### Analysis of elements contained in the metals by energy‐dispersive spectroscopy (EDS)

The metallic particles were observed with SEM and analysed qualitatively and quantitatively to determine their main components. EDS‐JEOL Model JSM‐6010PLUS was used for all analyses.

### Raman spectroscopy

Control and exposure to heavy metals of the previously lyophilized cells were used to analyse the formation of NPs using Raman spectrophotometry. Raman spectra of wetted samples on a silicon wafer (001) oriented surface were performed with a WITec Alpha300 microscope (Ulm, Germany) using a 633 nm laser for excitation. The integration time per Raman spectrum was between 10 and 60 s, which gives a sufficient signal‐to‐noise ratio without destroying the samples. The data were evaluated using the software program WITec project 2.10 (Ulm, Germany).

### X‐Ray powder diffraction (XRPD)

X‐Ray powder diffraction (XRPD) analysis was performed at the X‐ray diffraction beamline (XRD1) of the Elettra Synchrotron, Trieste, Italy (Lausi *et al*., 2015). Powder diffraction patterns were collected in transmission mode, at room temperature (25°C) with a monochromatic wavelength of 0.77491 Å (16 keV) and 200 × 200 μm^2^ spot size, using a Pilatus 2M hybrid‐pixel area detector. *Candida* cells exposed to precious and heavy metals were packed in 700‐μm diameter borosilicate capillaries (10 μm wall thickness). Blank samples were analysed in the same way, collecting data of lyophilized cells not exposed to heavy metals but treated with the same protocol (same growth parameters, washing and lyophilisation steps) as described by Cuéllar‐Cruz *et al*. (2017). Two‐dimensional powder patterns were integrated using Fit2D program (Hammersley, 2016), after preliminary calibration of the hardware setup, using a capillary filled with LaB_6_ standard reference powder (NIST 660a). Fluorescence spectra were recorded for all the samples, during diffraction data acquisition on a Silicon drift Amptek X‐123SDD detector, perpendicular to the X‐rays beam.

## Conflict of interest

None declared.

## Supporting information


**Fig. S1.** Cells of *C. albicans*,* C. dubliniensis,* and *C. glabrata* in presence of precious or heavy metals. Scale bar is indicated in each photomicrograph to show the size of the cells.

**Fig. S2.** Formation of gold nanoparticles by the *Candida* species in the presence of Au^3+^.

**Fig. S3**. Formation of silver nanoparticles by the *Candida* species in the presence of Ag^+^.

**Fig. S4**. Formation of mercury drops by the *Candida* species in the presence of Hg^2+^.

**Fig. S5**. Formation of lead nanoparticles by the *Candida* species in the presence of Pb^2+^.
