# Supplementary figures and images for: Bioreduction of precious and heavy metals by Candida species under oxidative stress conditions
Source: Microb Biotechnol. 2019 Jan 7;12(6):1164–79. doi: 10.1111/1751-7915.13364 (PMC6801149; doi:10.1111/1751-7915.13364)

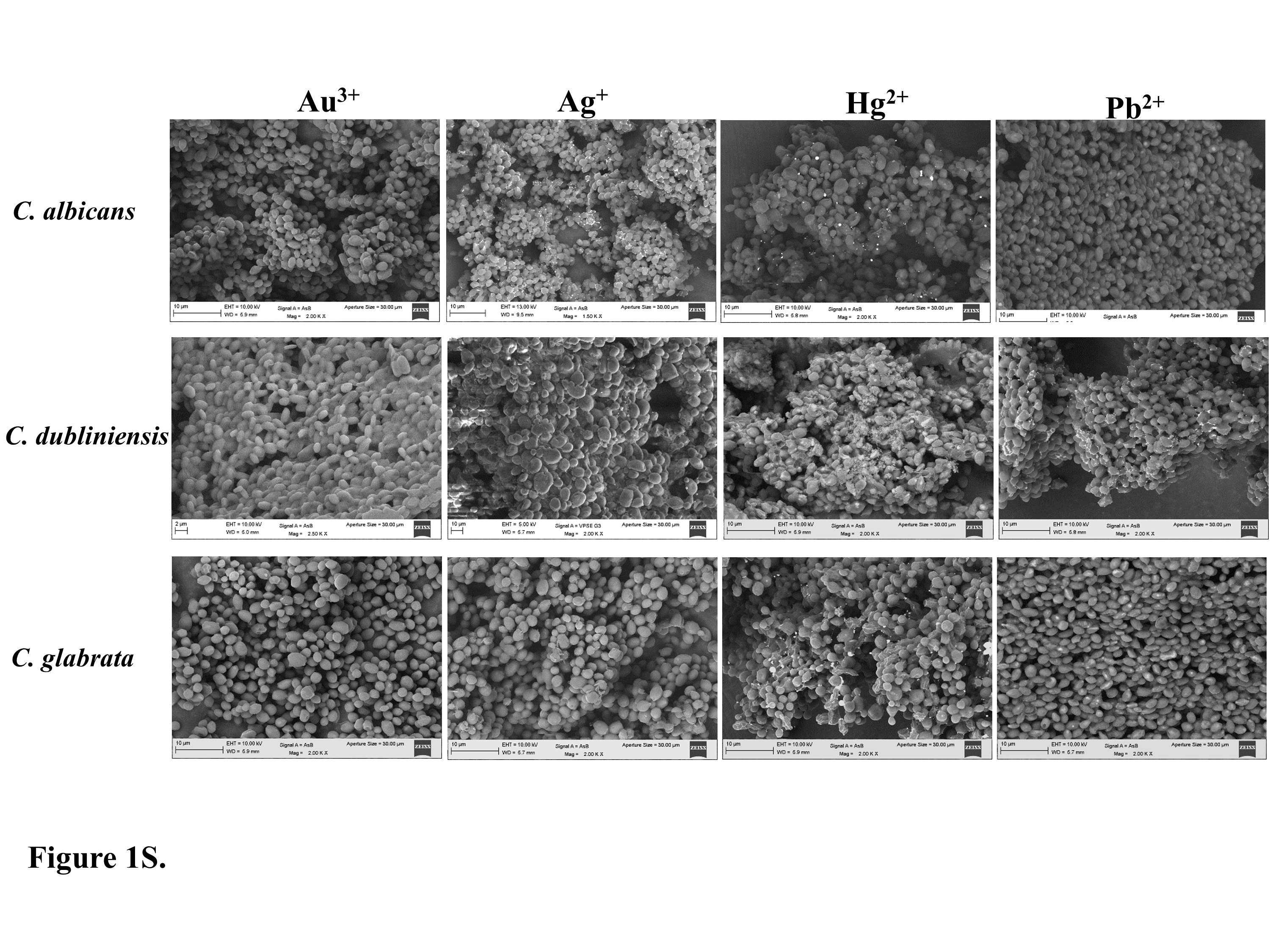

Supplement: Supplementary file 1 — Fig. S1. Cells of C. albicans, C. dubliniensis, and C. glabrata in presence of precious or heavy metals. Scale bar is indicated in each photomicrograph to show the size of the cells. [file MBT2-12-1164-s001.tif]

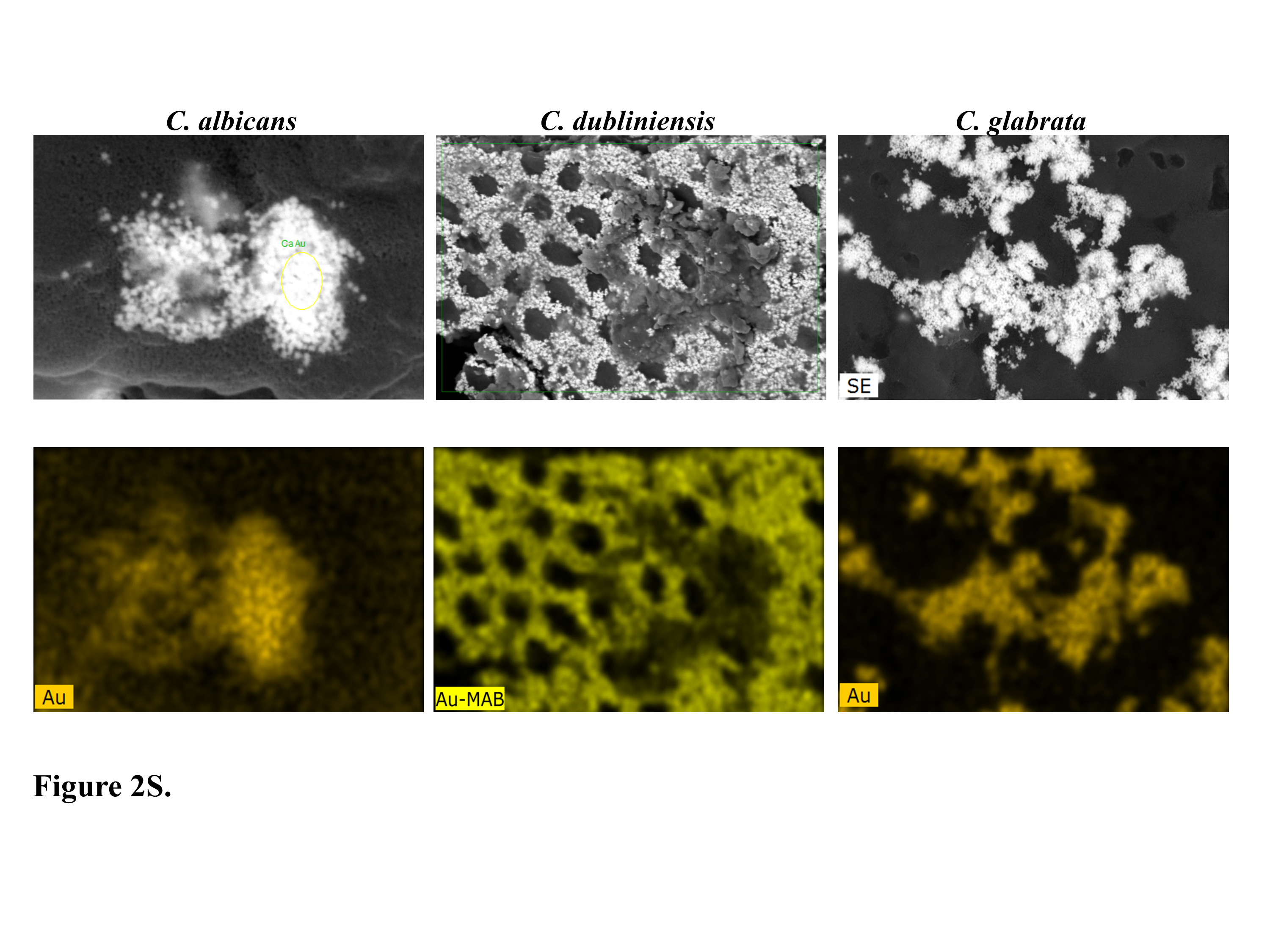

Supplement: Supplementary file 2 — Fig. S2. Formation of gold nanoparticles by the Candida species in the presence of Au3+. [file MBT2-12-1164-s002.tif]

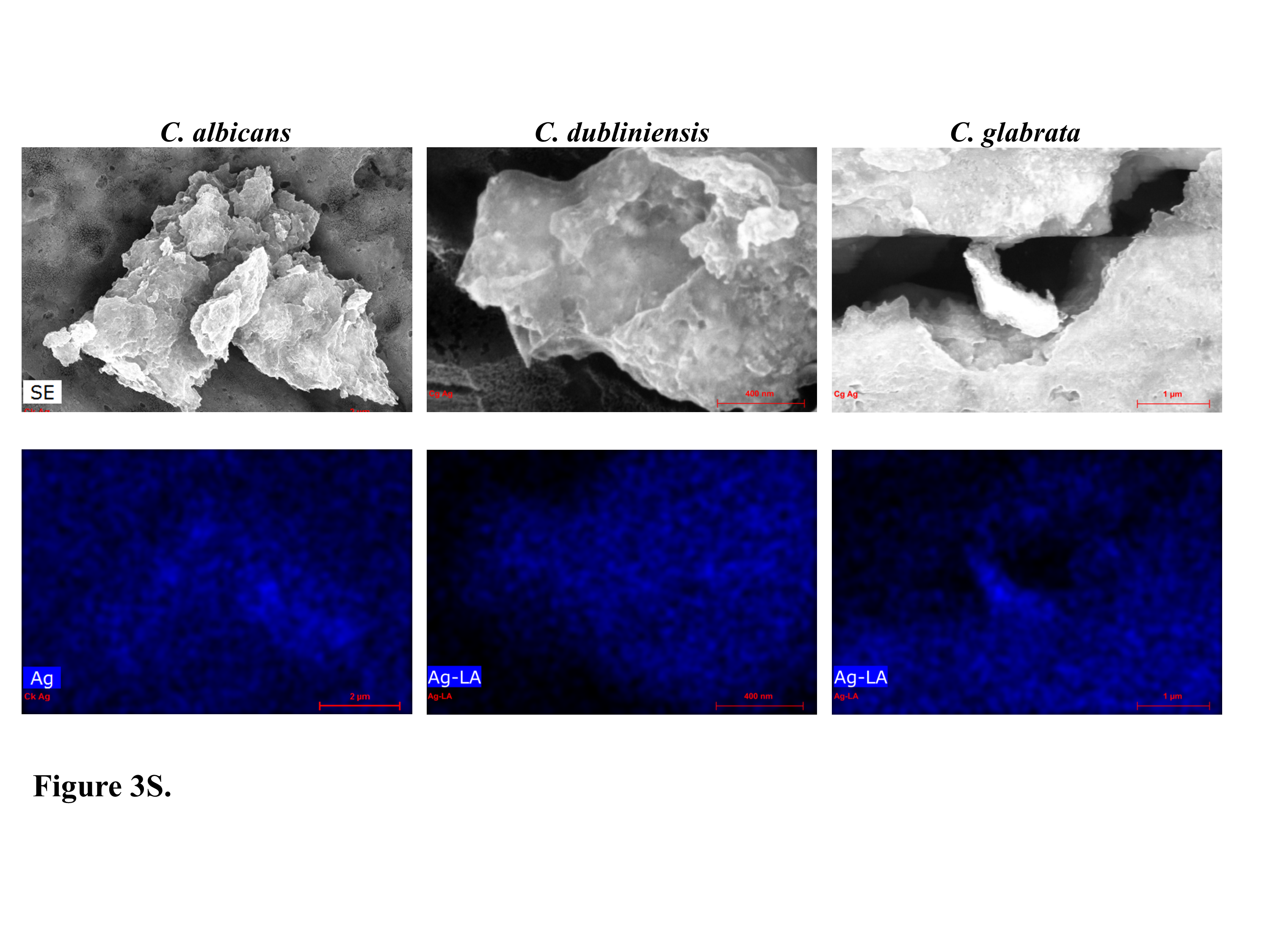

Supplement: Supplementary file 3 — Fig. S3. Formation of silver nanoparticles by the Candida species in the presence of Ag+. [file MBT2-12-1164-s003.tif]

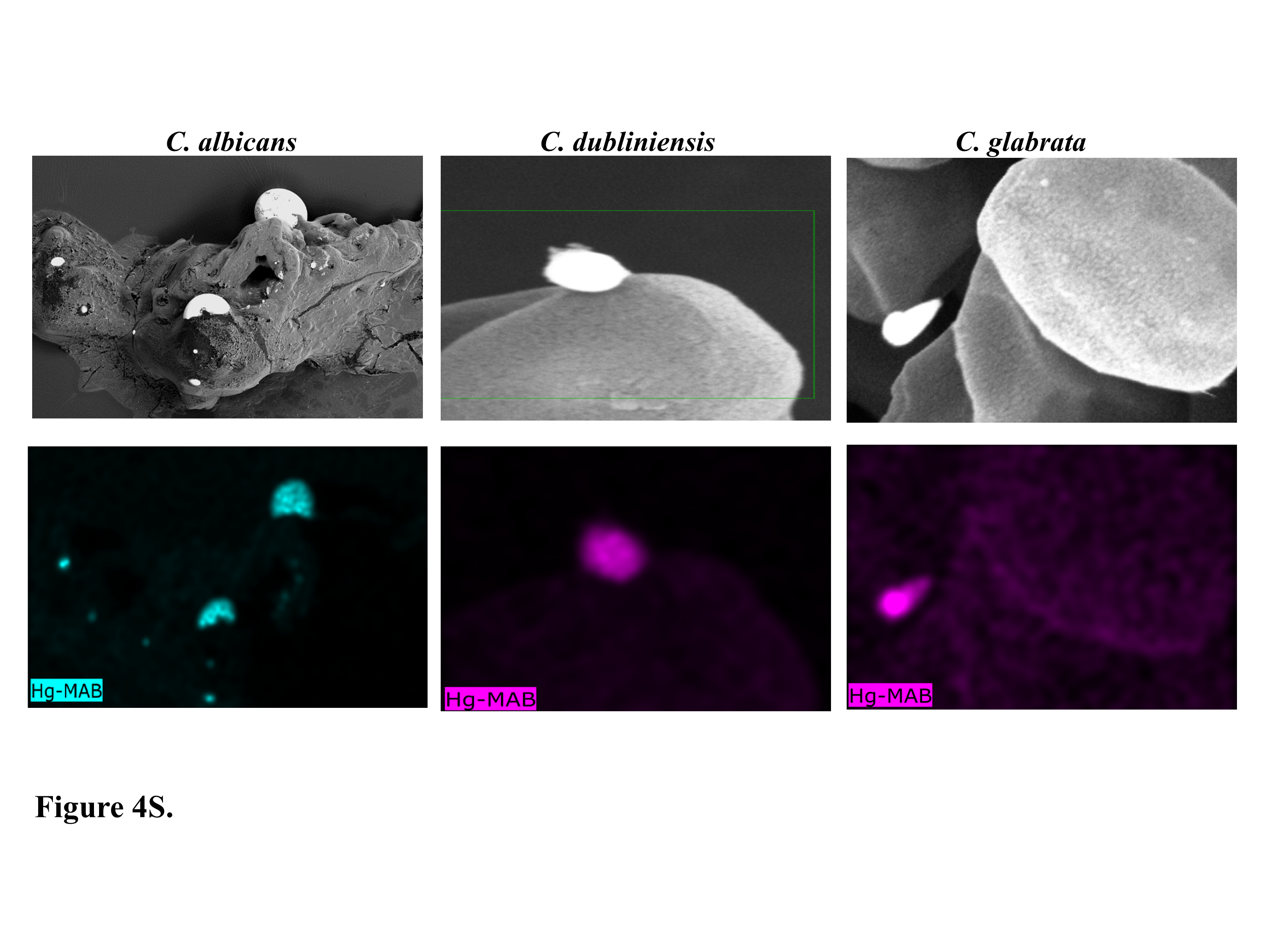

Supplement: Supplementary file 4 — Fig. S4. Formation of mercury drops by the Candida species in the presence of Hg2+. [file MBT2-12-1164-s004.tif]

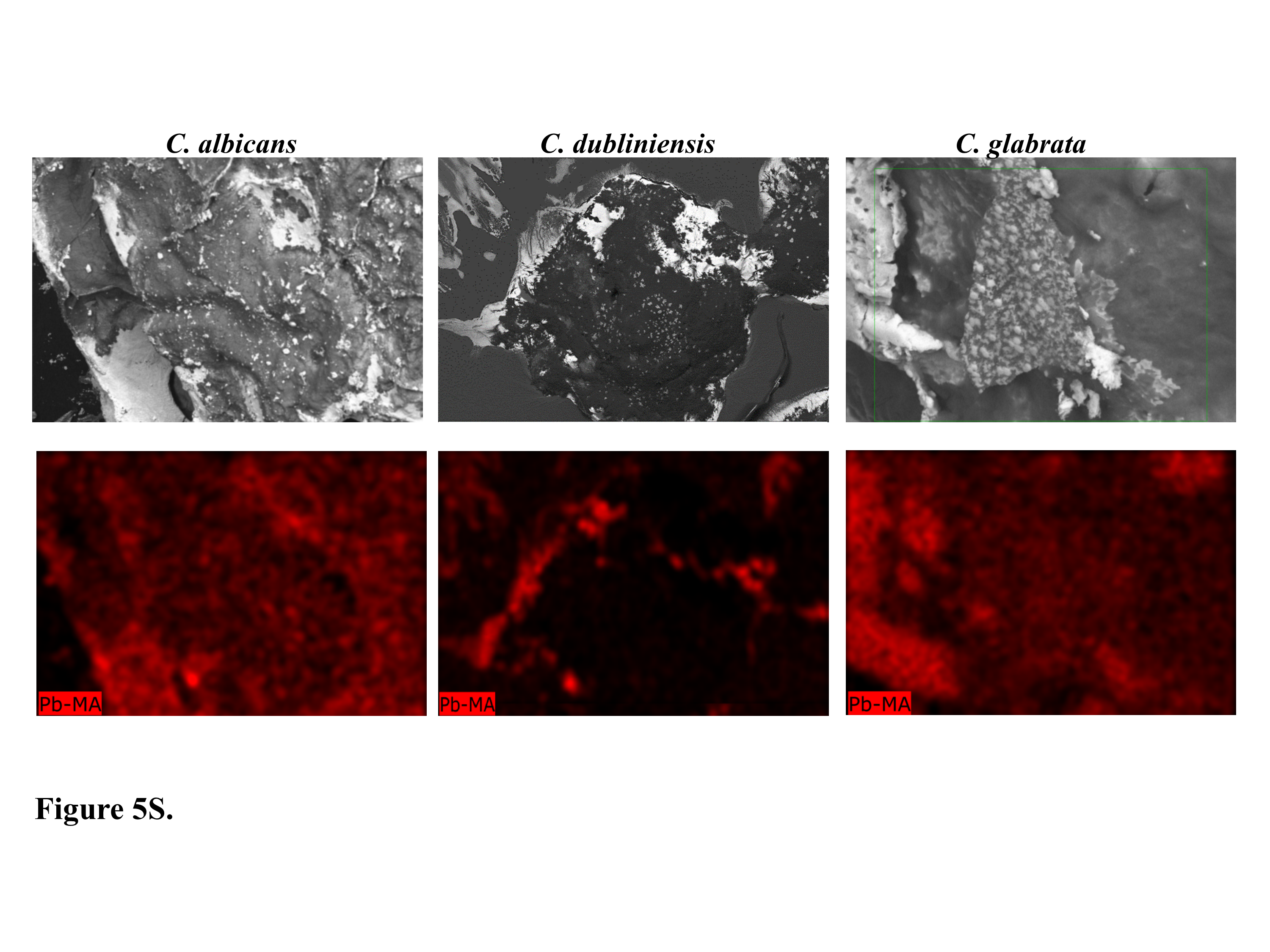

Supplement: Supplementary file 5 — Fig. S5. Formation of lead nanoparticles by the Candida species in the presence of Pb2+. [file MBT2-12-1164-s005.tif]
